# Reduced vertebrate diversity independent of spatial scale following feral swine invasions

**DOI:** 10.1002/ece3.5360

**Published:** 2019-06-14

**Authors:** Matthew R. Ivey, Michael Colvin, Bronson K. Strickland, Marcus A. Lashley

**Affiliations:** ^1^ Mississippi State University Mississippi State Mississippi

**Keywords:** agroecosystem, biodiversity, biological invasions, feral pig, *Sus scrofa*, vertebrate invasion

## Abstract

Biological invasions often have contrasting consequences with reports of invasions decreasing diversity at small scales and facilitating diversity at large scales. Thus, previous literature has concluded that invasions have a fundamental spatial scale‐dependent relationship with diversity. Whether the scale‐dependent effects apply to vertebrate invaders is questionable because studies consistently report that vertebrate invasions produce different outcomes than plant or invertebrate invasions. Namely, vertebrate invasions generally have a larger effect size on species richness and vertebrate invaders commonly cause extinction, whereas extinctions are rare following invertebrate or plant invasions. In an agroecosystem invaded by a non‐native ungulate (i.e., feral swine, *Sus scrofa*), we monitored species richness of native vertebrates in forest fragments ranging across four orders of magnitude in area. We tested three predictions of the scale‐dependence hypothesis: (a) Vertebrate species richness would positively increase with area, (b) the species richness y‐intercept would be lower when invaded, and (c) the rate of native species accumulation with area would be steeper when invaded. Indeed, native vertebrate richness increased with area and the species richness was 26% lower than should be expected when the invasive ungulate was present. However, there was no evidence that the relationship was scale dependent. Our data indicate the scale‐dependent effect of biological invasions may not apply to vertebrate invasions.

## 1 INTRODUCTION

Biological invasions are commonly regarded as one of the greatest threats to native biodiversity, particularly in island systems because they are discrete ecosystems with relatively little influence from immigration and emigration (e.g., Caujapé‐Castells et al., 2010; Kueffer et al., 2010; McCreless et al., 2016). However, biological invasions can have contrasting effects on native biodiversity. Most studies report negative effects of biological invasions on native species richness (Mollot, Pantel, & Romanuk, 2017). For example, introduced fire ants (*Solenopsis invicta*) reduced native ant diversity by 70% (Porter & Savignano, 1990), feral cats (*Felis catus*) are responsible for at least 14% of vertebrate extinctions on islands worldwide (Medina et al., 2011), and plant invasions in Mediterranean regions negatively impact native plant diversity (Gaertner, Breeyen, Hui, & Richardson, 2009). However, meta‐analyses of biological invasions also report that facilitation of species richness by invaders is common (Gaertner et al., 2009; Rodriguez, 2006). For example, zebra mussels (*Dreissena polymorpha*) decrease native bivalve richness substantially, but facilitate invertebrate biodiversity when present (Bially & Macisaac, 2000). Similarly, invasive European green crabs (*Carcinus maenas*) prey upon a native clam species decreasing its dominance in the community, which resulted in greater diversity of benthic invertebrates (Grosholz, 2005; Grosholz et al., 2000). Another example was documented with a non‐native toad (*Bufo marinus*) that facilitated native anuran prey through the reduction of native predatory anuran populations (Crossland, 2000). These examples demonstrate that the consequences of biological invasions to biodiversity can be variable and sometimes are context dependent even within a species of invader.

Scale‐dependent effects of biological invasions have been proposed as a universal explanation of contrasting effects of biological invasions on biodiversity (Powell, Chase, & Knight, 2013). For example, Powell et al. (2013) reported plant invasions negatively affected plant diversity at small scales but had no effect at large scales. Similarly, Altieri, van Wesenbeeck, Bertness, and Silliman (2010) reported a positive relationship only at the large scale between invasibility and biodiversity of an invertebrate invasion. This positive relationship was explained by facilitation cascades resulting from small‐scale positive interactions across trophic levels. However, dissimilarities between the effects of nonvertebrate and vertebrate biological invasions on islands bring into question whether the scale‐dependent pattern reported by Powell et al. (2013), Altieri et al. (2010), and many others also applies to vertebrate biological invasions. That is, on islands, vertebrate invasions generally have a larger effect size than nonvertebrate invaders (Mollot et al., 2017), and plant invasions rarely cause extinctions of plant species (Sax & Gaines, 2008), but vertebrate invaders are the leading cause of vertebrate extinctions (Bellard, Cassey, & Blackburn, 2016; Clavero & García‐Berthou, 2005; Doherty, Glen, Nimmo, Ritchie, & Dickman, 2016). More importantly from the perspective of the scale‐dependence hypothesis, biodiversity facilitation is most commonly reported in nonvertebrate invasions (Rodriguez, 2006), which may indicate facilitation at large scales occurs less often following vertebrate invasions or may simply be an artifact of taxonomic bias in observations (Sax & Gaines, 2008).

The species–area relationship is fundamental in ecology (May, 1975, Connor and McCoy, 1979; Rosenzweig, 1995) and is a widely used tool to predict declining diversity (Primack, 2006). Because species richness has a predictable positive relationship with area, this tool could be useful to evaluate how biological invasions affect native species richness (Sax & Gaines, 2008). Because islands can be relatively isolated replicates of discrete ecosystems, they can serve as good model systems for understanding how invasions affect native species (Patino et al., 2017). Thus, pairing the species–area relationship with similar islands that vary in size could allow a direct method of quantifying the effects of species invasions by establishing a baseline in species richness in the per area species richness of islands and then comparing the per area species richness of invaded islands to that baseline. Agricultural ecosystems create islands through forest fragmentation in which plant species–area relationships have been documented (Giladi, Ziv, May, & Jeltsch, 2011). Thus, because forest fragments may vary substantially in size, they may also serve as a good model system to evaluate how invasive vertebrate species affect native vertebrate species–area relationships. Moreover, feral swine (*Sus scrofa*) are one of the most invasive vertebrate species in the world with one of the widest geographic ranges of any large mammal (Barrios‐Garcia & Ballari, 2012; Lowe, Browne, Boudjelas, & De Poorter, 2000), have become a nuisance in many agricultural ecosystems (Anderson, Slootmaker, Harper, Holderieath, & Shwiff, 2016), and have a high probability of competing with native vertebrates (O'Brien et al., 2019). Also, feral swine commonly have a strong negative influence on native species via exploitative competition and predation (Bevins, Pedersen, Lutman, Gidlewski, & Deliberto, 2014; Doherty et al., 2016; Graves, 1984; Gurevitch & Padilla, 2004; Mollot et al., 2017; Yang, Bastow, Spence, & Wright, 2008). Thus, forest fragments invaded by feral swine in agricultural ecosystems should allow a good study model to test the scale‐dependence hypothesis following a vertebrate invasion.

In forest fragments recently invaded by feral swine within agroecosystems of the Mississippi Alluvial Valley and utilizing the fundamental species–area relationship, we tested three predictions to evaluate the hypothesis that vertebrate biological invasions have a scale‐dependent effect on vertebrate species richness. We predicted the number of native species detected would increase with increasing area (i.e., follow predictions of the species–area relationship), that we would detect less native vertebrates than expected in relatively small forest fragments that were invaded by feral swine, and that invaded forest fragments would have a steeper slope of accumulation with increasing area (i.e., scale‐dependent effect on species richness, Powell et al., 2013). To test these predictions, we monitored species richness of vertebrate communities detectable via camera trapping in forest fragments ranging across four orders of magnitude in area with and without feral swine.

## 2 METHODS

**Figure 1:**
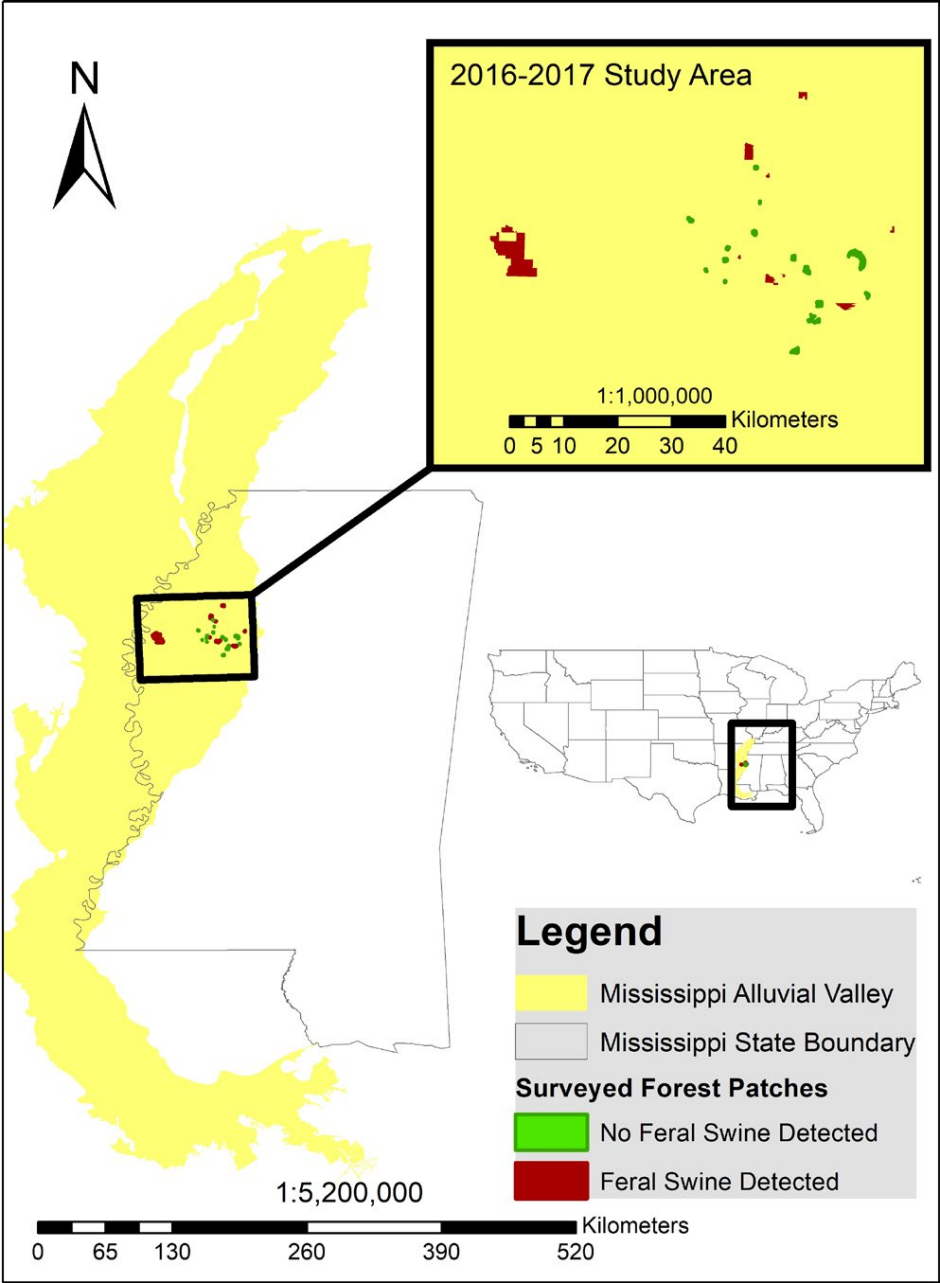
The spatial distribution of forest fragments sampled between February and October of 2016 and 2017 to determine the effects of feral swine invasion on native vertebrate species richness. Green polygons represent forest fragments where feral swine were not detected, and red polygons indicate forest fragments where feral swine were detected via camera trapping. Located in the Mississippi Alluvial Valley in Mississippi, USA

We monitored vertebrate species richness within forest fragments in agroecosystems of the Mississippi River Alluvial Valley in Mississippi, USA (33°40′18.0′′N 90°29′57.8′′W, Figure 1). Nearly 75% of the historic bottomland hardwood forests found in the Mississippi Alluvial Valley have been converted into agriculture, with the remaining forest fragmented into over 38,000 fragments larger than 2 ha (Twedt & Loesch, 1999) in which the dominant plant community was bottomland hardwood forests. We established systematically random grids of sample points across each of 36 forest fragments a priori using a 20‐hectare grid overlay in ArcMap 10.3.1 (ESRI, 2015). We used paired opposing camera traps (Rovero & Marshall, 2009) approximately 0.75 meters above the ground and approximately 6 m apart at a density of one pair per 20 hectares. The 36 forest fragments ranged from 3 to 4,000 hectares in area**.** We used the trail‐targeting method to strategically place camera traps near animal activity to maximize detection without the use of attractants (Kays et al., 2009; Kolowski & Forrester., 2017; Rovero & Marshall, 2009; Tobler, Carrillo‐Percastegui, Leite Pitman, Mares, & Powell, 2008). Area of fragments was calculated within ArcMap 10.3.1 (ESRI, 2015). Any fragment with at least one feral swine detection was considered invaded. Similarly, all species detected on at least one occasion were included in the estimate of species richness for the respective fragment. We acknowledge that only counting species detectable by camera traps does not represent all species within a fragment; however, using a subset of species richness has been determined to be representative of the true species richness and statistically sound for ecological studies on biodiversity (Vellend, Lilley, & Starzomski, 2008). Between February and October 2016 and 2017, we sampled each forest fragment for 30 days. To ensure sampling events were long enough in duration to detect the majority of species present based on species accumulation rates, species accumulation curves for each fragment were created within R Studio version 1.0.136 using the “vegan” package and “specaccum” function set to random with 1,000 permutations. Results from that analysis confirmed that species accumulation plateaued around 10 days on average (Figure S1). Thus, the 30‐day period was three times longer than the time needed to detect the majority of species detectable via camera trapping within a forest fragment. We analyzed the data both with and without nine‐banded armadillo (*Dasypus novemcinctus*) in species richness due to their potentially human‐facilitated range expansion in the United States (Humphrey, 1974; Taulman & Robbins, 1996). While the armadillos are historically non‐native, they are now a naturalized species. So, it could be argued that they should or should not be included in our analysis based on their status. Thus, in the interest of transparency, we modeled species richness with and without the inclusion of armadillos. Two linear models were fit within Program R (1: ln(SpeciesRichness) ~ ln(Area), 2: ln(SpeciesRichness) ~ ln(Area) + Swine Occurrence) to test the predictions that (a) species richness would increase with increasing area, (b) species richness would be suppressed within invaded fragments. A third model was fit (ln(SpeciesRichness) ~ ln(Area) + Swine Occurrence + ln(Area) × Swine Occurrence), testing our prediction that invaded fragments would have a higher species accumulation rate (steeper slope) than noninvaded (i.e., significant interaction term). We evaluated which of these three competing nested models best supported the data using a likelihood ratio test (Hilborn & Mangel, 1997).

## 3 RESULTS

**Figure 2:**
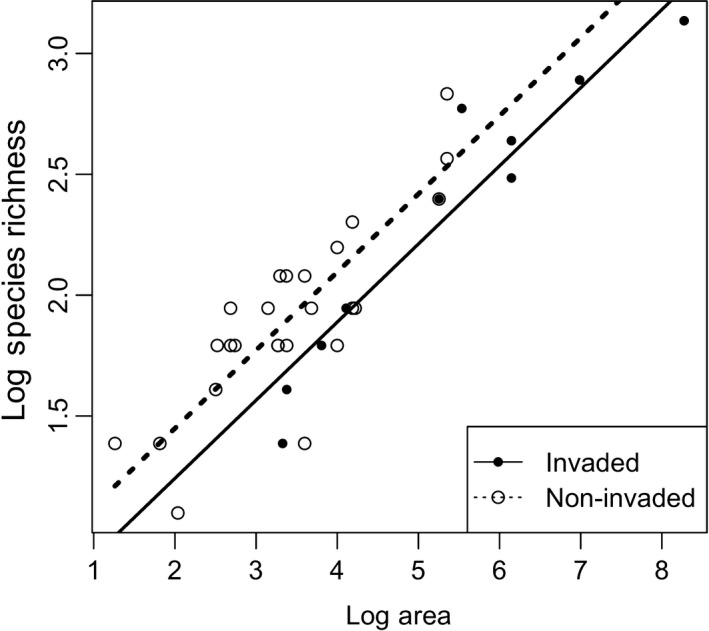
Log–Log relationship between species richness and forest fragment area in the Mississippi Alluvial Valley invaded (solid line and solid points) and absent (broken line and hollow points) of feral swine. Lines indicate that area has a positive effect on species richness, and species richness was 26% lower when invaded by swine and a lack of scale dependence (i.e., parallel slopes) in the effects of the invasion. In this analysis, naturalized non‐native nine‐banded armadillos were not included in the species richness

We detected 41 unique species, 39 of which were vertebrates used in our models, with estimated species richness ranging from 4 to 26 species in a single forest fragment (Table S1). We detected feral swine in 11 out of 36 fragments ranging from 28 to 4,000 hectares in size. Based on the likelihood ratio test, (ln(SpeciesRichness) ~ ln(Area) + Swine Occurrence) was the top model (*p* = 0.028; Table 1). This model revealed a strong positive effect of area on species richness (*R* ^2^ = 0.83; *p* < 0.001, Figure 2). We initially considered the time of year of sampling as a potentially influential factor when estimating species richness but adding the timing of sampling as a covariate in this model was not significant (Figure S2). Species richness per area of feral swine‐invaded forest fragments was 26% lower (*p* = 0.026) than uninvaded forest fragments (17% lower when including the naturalized armadillos in the species richness; *p* = 0.029; Figure S3). The effect of feral swine was not scale dependent as indicated by the lack of interaction between area and invasion (Model 3: ln(SpeciesRichness) ~ ln(Area) + Swine Occurrence + ln(Area) × Swine Occurrence; *p* = 0.43). Thus, species richness estimates were 26% lower in forest fragments where we detected feral swine than should have been expected based on the area of the fragments and that relationship held true across the range of fragment area.

**Table 1:** Comparison of models selected to test the scale‐dependence hypothesis in an agroecosystem invaded by feral swine in the Mississippi Alluvial Valley, MS, USA

Model	*k*	Log‐likelihood	AIC	*R*‐squared	Adj. *R*‐Squared
SRLog ~ AreaLog	2	5.125	−4.251	0.796	0.790
SRLog ~ AreaLog + Pig	3	7.864	−7.728	0.825	0.814
SRLog ~ AreaLog × Pig	4	8.047	−6.094	0.827	0.811

## 4 DISCUSSION

Native vertebrate species richness was 26% lower than expected based on patch area when swine were present. Although our study was not designed to determine the mechanism by which species richness suppression occurred, our observations are consistent with the average declines observed in other biological invasions (i.e., 21%–27%; Mollot et al., 2017). Feral swine have a wide dietary breadth and disturb habitat structure through rooting and wallowing (Barrios‐Garcia & Ballari, 2012; Graves, 1984). Thus, predation, habitat degradation, interference, or exploitative competition could be responsible for the observed decrease. Because the effect size of predator invasions on richness is generally on the larger end of the scale (Mollot et al., 2017), our observations may indicate predation risk as the more likely driver of declines reported herein though habitat disturbance and competition may also be contributing factors. Also, it is possible that many of the native species simply avoided areas where feral swine were present as has been documented elsewhere (O'Brien et al., 2019). However, our results should be interpreted with caution because we did not establish causality in this study and could not rule out that swine invasion probability of a fragment did not correlate with some other factors making it less species rich than should be expected. That said, we believe it is unlikely that swine invasions correlate with another causative mechanism especially given the foraging habits of swine coupled with consistencies observed in other biological invasions and the fact that all areas sampled in this study had relatively similar vegetation types.

The lack of scale‐dependent effects of feral swine on species richness we observed here brings into question whether the scale‐dependence hypothesis is a universal consequence of biological invasions. In fact, we were unable to find any examples of vertebrate invasions having scale‐dependent effects on native species richness even though this relationship commonly has been demonstrated when the invader is a plant (e.g., Fridley, Brown, & Bruno, 2004, Davies et al., 2005, Powell et al., 2013) or invertebrate (e.g., Mayer, Keats, Rudstam, & Mills, 2002, Altieri et al., 2010, Pintor & Sih, 2011). Those scale‐dependent effects were likely a function of facilitation (Rodriguez, 2006). Thus, we may not have detected scale dependence because feral swine may not facilitate native vertebrates at any scale. However, a scale‐dependent effect could be present if feral swine facilitated species richness of taxa not detectable via camera trapping, as they have been documented to facilitate some plant and animal species as a result of biopedturbation (Barrios‐Garcia & Ballari, 2012; Baruzzi & Krofel, 2017). Similarly, scale dependence could be a function of changing density of the invader across scale, but feral swine abundance scaled linearly (i.e., density was stable) across fragment size in this study area (Ivey 2018).

More than 70% of the world's forests are within 1 km of forest edge, which is within the range of being influenced by human activity (Haddad et al., 2015). With increased human influence, habitat fragmentation will likely become more common (Tilman et al., 2001), and increases in fragmentation will affect species differently. For example, generalists are less sensitive to habitat fragmentation (Keinath et al., 2017), and fragmentation may even favor invasion by habitat generalists (Marvier, Kareiva, & Neubert, 2004). Because habitat fragmentation is likely to increase (Sala et al., 2000), vertebrate invaders are not as vulnerable to fragmentation (Keinath et al., 2017), and invasions may decrease species richness (Blackburn, Cassey, Duncan, Evans, & Gaston, 2004), biological invasions coupled with habitat fragmentation may be a nonlinearly increasing threat to biodiversity (Haddad et al., 2015).

Eradication of invasive species may be a necessary step to maintain biodiversity (Courchamp, Chapuis, & Pascal, 2003; Glen et al., 2013). Several examples exist where eradication of invasive species resulted in an increase in native species richness or endangered species recovery. For example, eradication of red (*Vulpes vulpes*) and arctic foxes (*Vulpes lagopus*) from Alaskan islands resulted in recovery of endangered Aleutian Canada geese (*Branta hutchinsii leucopareia*; Byrd, 1998). In a meta‐analysis of invasive mammal eradications, Jones et al. (2016) documented 123 recolonizations of formerly extirpated native species following invader eradication. However, there has been little success in large‐scale eradication efforts with exception of the eradication of Norway rats (*Rattus norvegicus*) in New Zealand from large islands (Clout & Veitch, 2002). Most case studies have demonstrated eradication success on small islands and local scales (Zavaleta, Hobbs, & Mooney, 2001). In fact, 78% of successful rodent eradications were on islands < 100 ha in area (Howald et al., 2007). Interestingly, populations on small islands that are more isolated have a higher probability of extinction—a fundamental of the equilibrium theory (MacArthur & Wilson, 1967). Thus, this fundamental concept may be useful to predict the probability of success in invasive species eradication efforts. If the island species–area relationships with extinction probability hold true in fragmented terrestrial landscapes, eradication efforts focused on small fragments with the most vulnerable populations may be most effective. With isolation as a secondary contributing factor, increasing isolation of larger islands by eradicating small island populations first may also be an effective strategy for eradication. Future research is needed to understand whether island biogeography theory and species–area relationships can be useful in invasive species eradication efforts and the conservation of native biodiversity in a broad range of island ecosystems.

## CONFLICT OF INTEREST

The authors declare no competing interests.

## AUTHOR CONTRIBUTIONS

MI and ML designed the study. MI performed the field work and camera trapping surveys. MI, MC, and ML analyzed the data. MI wrote the manuscript; all authors contributed to manuscript editing and approved the final version.

## Data Availability

Data used in this manuscript are included in the supplementary files (Table S2).
