# Supplementary material for: Reduced vertebrate diversity independent of spatial scale following feral swine invasions
Source: Ecol Evol. 2019 Jun 14;9(13):7761–7. doi: 10.1002/ece3.5360 (PMC6635915; doi:10.1002/ece3.5360)
Supplement: Supplementary file 1 [file ECE3-9-7761-s001.docx]

| Table S1. Species detected via camera trapping in 36 forest fragments ranging from 3-4000ha in area in the Mississippi Alluvial Valley, Mississippi, USA. | |
| --- | --- |
| **Common Name** | **Scientific Name** |
| **White-footed Mouse** | *Peromyscus leucopus* |
| **Wood Rat** | *Neotoma floridana* |
| **Eastern Chipmunk** | *Tamias striatus* |
| **Grey Squirrel** | *Sciurus carolinensis* |
| **Fox Squirrel** | *Sciurus niger* |
| **Southern Flying Squirrel** | *Glaucomys volans* |
| **Eastern Cottontail** | *Sylvilagus floridanus* |
| **Raccoon** | *Procyon lotor* |
| **Virginia Opossum** | *Didelphis virginiana* |
| **Striped Skunk** | *Mephitis mephitis* |
| **River Otter** | *Lontra canadensis* |
| **Grey Fox** | *Urocyon cinereoargenteus* |
| **Bobcat** | *Lynx rufus* |
| **Coyote** | *Canis latrans* |
| **Feral Pig** | *Sus scrofa* |
| **Nine-banded Armadillo** | *Dasypus novemcinctus* |
| **American Robin** | *Turdus migratorius* |
| **Brown Thrasher** | *Toxostoma rufum* |
| **Common Grackle** | *Quiscalus quiscula* |
| **Blue Jay** | *Cyanocitta cristata* |
| **Northern Cardinal** | *Cardinalis cardinalis* |
| **Tufted Titmouse** | *Baeolophus bicolor* |
| **Carolina Chickadee** | *Poecile carolinensis* |
| **Louisiana Waterthrush** | *Parkesia motacilla* |
| **Chipping Sparrow** | *Spizella passerina* |
| **Eastern Phoebe** | *Sayornis phoebe* |
| **Mourning Dove** | *Zenaida macroura* |
| **Red-headed Woodpecker** | *Melanerpes erythrocephalus* |
| **Red-bellied Woodpecker** | *Melanerpes carolinus* |
| **Pileated Woodpecker** | *Dryocopus pileatus* |
| **Northern Flicker** | *Colaptes auratus* |
| **Barred Owl** | *Strix varia* |
| **Red-shouldered Hawk** | *Buteo lineatus* |
| **Turkey Vulture** | *Cathartes aura* |
| **Great Blue Heron** | *Ardea herodias* |
| **Wood Duck** | *Aix sponsa* |
| **Three-toed Box Turtle** | *Terrapene carolina triunguis* |
| **Diamondback Terrapin** | *Malaclemys terrapin* |
| **Black Rat Snake** | *Pantherophis obsoletus* |

| Table S2. Data set generated via camera trapping to test the scale dependence hypothesis with feral swine in forest fragments of the Mississippi Alluvial Valley, MS, USA. | | | | | |
| --- | --- | --- | --- | --- | --- |
| **Vertebrate Species Richness** | **Feral Swine Present** | **Year** | **AreaLog** | **NDLog** | **SRLog** |
| 17 | No | 2016 | 5.35264 | 2.977059 | 2.833213 |
| 8 | No | 2016 | 3.373312178 | 4.025709 | 2.079442 |
| 7 | No | 2016 | 3.147336858 | 4.216429 | 1.94591 |
| 6 | No | 2016 | 3.269721019 | 4.437213 | 1.791759 |
| 10 | No | 2016 | 4.186923744 | 4.695404 | 2.302585 |
| 9 | No | 2016 | 3.999704158 | 5.694409 | 2.197225 |
| 4 | No | 2016 | 3.598927394 | 6.117134 | 1.386294 |
| 6 | No | 2016 | 2.685736424 | 6.87133 | 1.791759 |
| 3 | No | 2016 | 2.036664512 | 7.034195 | 1.098612 |
| 12 | Yes | 2016 | 6.146282147 | 4.172632 | 2.484907 |
| 11 | Yes | 2016 | 5.254245305 | 4.483567 | 2.397895 |
| 7 | Yes | 2016 | 4.109873364 | 5.238493 | 1.94591 |
| 18 | Yes | 2016 | 6.986724488 | 6.538889 | 2.890372 |
| 16 | Yes | 2016 | 5.534965322 | 6.821954 | 2.772589 |
| 5 | Yes | 2016 | 3.377792273 | 7.026352 | 1.609438 |
| 4 | Yes | 2016 | 3.327299644 | 7.081245 | 1.386294 |
| 6 | No | 2017 | 2.521800941 | 5.960982 | 1.791759 |
| 8 | No | 2017 | 3.294725137 | 5.617164 | 2.079442 |
| 5 | No | 2017 | 2.503073954 | 5.617164 | 1.609438 |
| 6 | No | 2017 | 3.269721019 | 4.437213 | 1.791759 |
| 7 | No | 2017 | 2.685736424 | 6.87133 | 1.94591 |
| 8 | No | 2017 | 3.598927394 | 6.117134 | 2.079442 |
| 6 | No | 2017 | 2.739548868 | 6.560725 | 1.791759 |
| 7 | Yes | 2017 | 4.109873364 | 5.238493 | 1.94591 |
| 6 | Yes | 2017 | 3.806440243 | 5.526034 | 1.791759 |
| 6 | No | 2017 | 3.377792273 | 7.026352 | 1.791759 |
| 6 | No | 2017 | 3.999704158 | 5.694409 | 1.791759 |
| 7 | No | 2017 | 3.681955539 | 6.054369 | 1.94591 |
| 4 | No | 2017 | 1.261297871 | 5.635646 | 1.386294 |
| 7 | No | 2017 | 4.186923744 | 4.695404 | 1.94591 |
| 4 | No | 2017 | 1.814824742 | 5.863603 | 1.386294 |
| 7 | No | 2017 | 4.221270856 | 6.696454 | 1.94591 |
| 13 | No | 2017 | 5.352639818 | 2.977059 | 2.564949 |
| 11 | No | 2017 | 5.254245305 | 4.483567 | 2.397895 |
| 23 | Yes | 2017 | 8.274308204 | 4.60517 | 3.135494 |
| 14 | Yes | 2017 | 6.146282147 | 4.172632 | 2.639057 |
