# Supplementary material for: Reduced vertebrate diversity independent of spatial scale following feral swine invasions
Source: Ecol Evol. 2019 Jun 14;9(13):7761–7. doi: 10.1002/ece3.5360 (PMC6635915; doi:10.1002/ece3.5360)
Supplement: Supplementary file 2 [file ECE3-9-7761-s002.docx]

*Appendix S1: Reduced vertebrate diversity independent of spatial scale following feral swine invasions*

**Figure S1.** Species accumulation over time for 36 forest fragments sampled to determine the effects of feral swine (*Sus scrofa*) on species richness. Most species were detected within 14 days in most fragments indicating our 30-day sampling period was sufficient to estimate species richness. The lines depict species accumulation and the grey margins represent the number of possible species to detect (derived from 1000 random permutations within Program R).


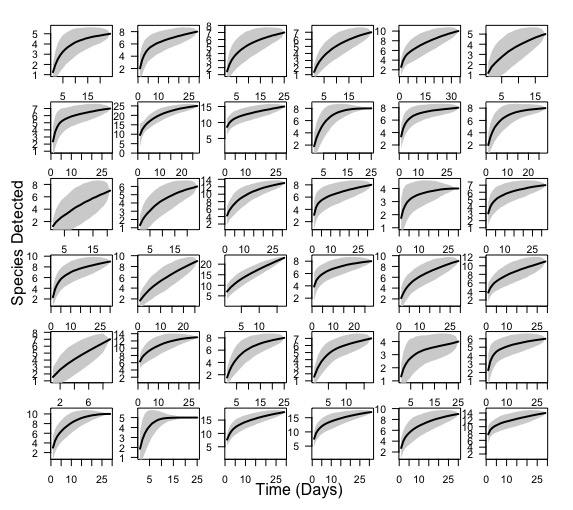


*Appendix S1: Reduced vertebrate diversity independent of spatial scale following feral swine invasions*

**Figure S2.** Relationship between Species per Area detected and Sample Time of camera trapping surveys in forest fragments invaded and non-invaded by feral swine (*Sus scrofa)* in the Mississippi Alluvial Valley. Timing of sampling was not a significant explanatory variable, only increasing the R2 value of the model by less than 1%.

*
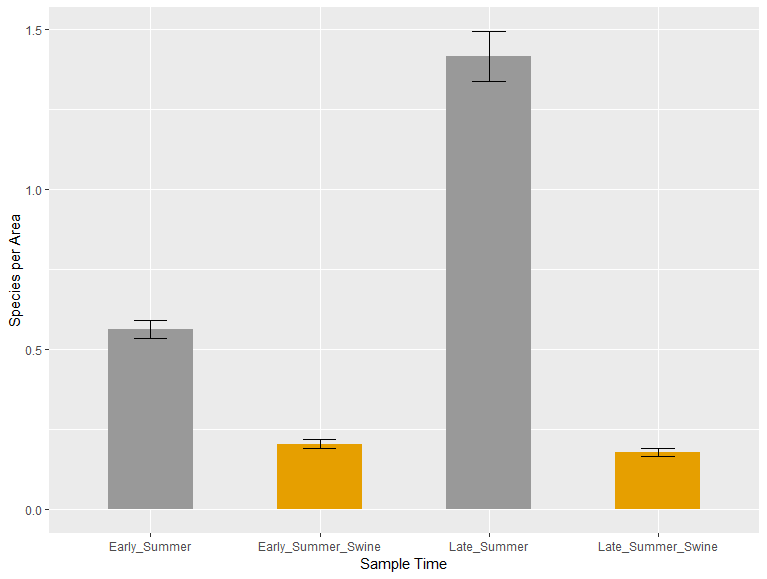
*

*Appendix S1: Reduced vertebrate diversity independent of spatial scale following feral swine invasions*

**Figure S3.** Log-Log relationship between species richness and area for forest fragments in the Mississippi Alluvial Valley invaded and absent of feral swine. Lines indicate that area has a positive effect on species richness, species richness was 17% lower when invaded by swine, and a lack of scale-dependence (i.e. increased slope when invaded) in the effects of the invasion. In this analysis, naturalized non-native nine-banded armadillos were included in species richness.

**
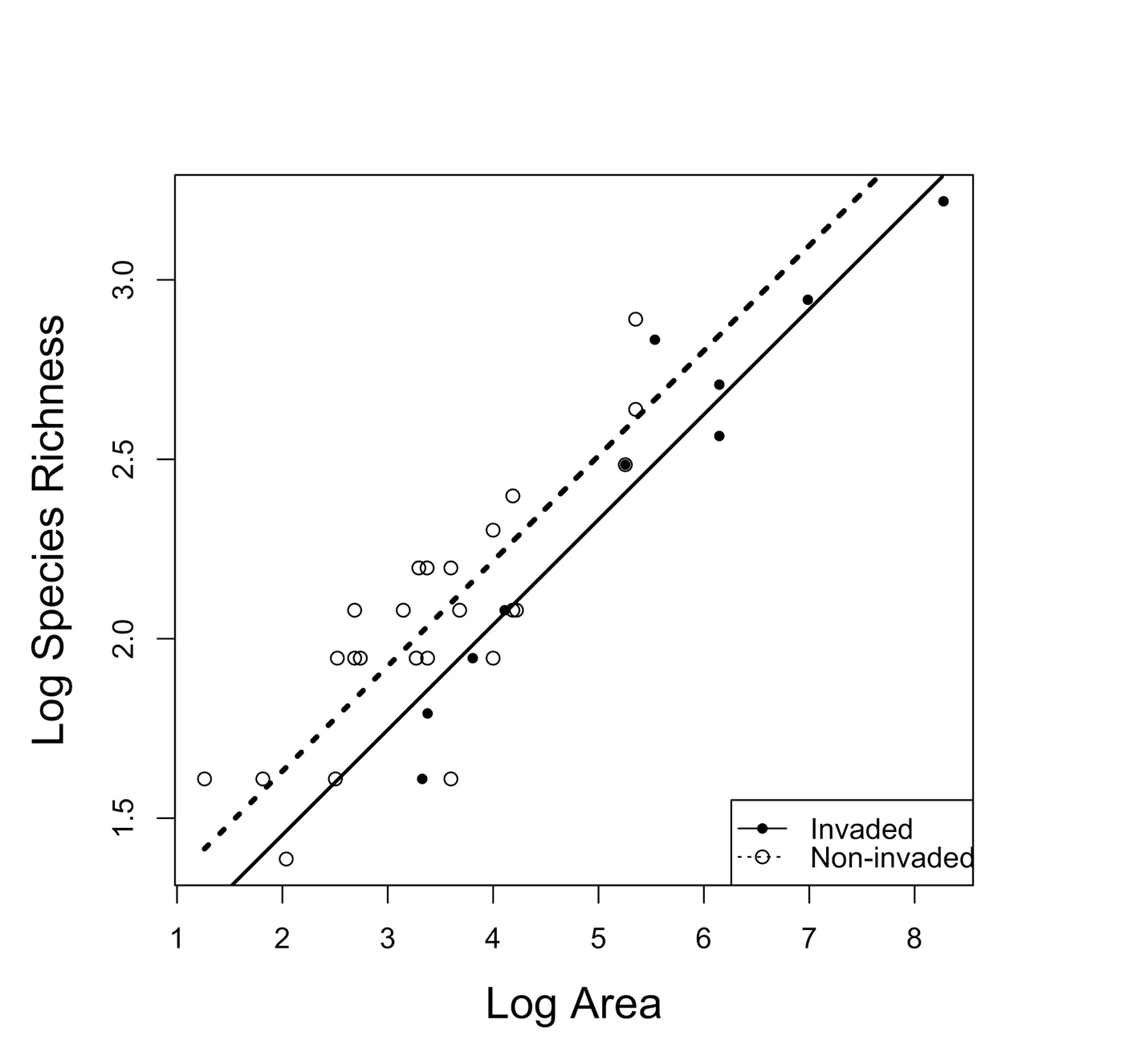
**
